# SGP-C: A Broad Host Range Temperate Bacteriophage; Against *Salmonella gallinarum*

**DOI:** 10.3389/fmicb.2021.768931

**Published:** 2022-01-12

**Authors:** Syeda Zainab Ilyas, Hafsa Tariq, Abdul Basit, Hamza Tahir, Zulquernain Haider, Shafiq ur Rehman

**Affiliations:** Institute of Microbiology and Molecular Genetics, University of the Punjab, Lahore, Pakistan

**Keywords:** *Salmonella gallinarum*, temperate phage, lysogens, superinfection immunity, next generation sequencing, repressor gene

## Abstract

*Salmonella gallinarum* is a poultry restricted-pathogen causing fowl-typhoid disease in adult birds with mortality rates up-to 80% and exhibit resistance against commonly used antibiotics. In this current study, a temperate broad host range bacteriophage SGP-C was isolated against *S. gallinarum* from poultry digesta. It showed infection ability in all the 15 tested field strains of *S. gallinarum*. The SGP-C phage produced circular, turbid plaques with alternate rings. Its optimum activity was observed at pH 7.0 and 37–42°C, with a latent period of 45 min and burst size of 187 virions/bacterial cell. The SGP-C lysogens, *SGPC-L5* and *SGPC-L6* exhibited super-infection immunity against the same phage, an already reported feature of lysogens. A virulence index of 0.5 and 0.001 as MV50 of SGP-C suggests its moderate virulence. The genome of SGP-C found circular double stranded DNA of 42 Kbp with 50.04% GC content, which encodes 63 ORFs. The presence of repressor gene at ORF49, and absence of tRNA sequence in SGP-C genome indicates its lysogenic nature. Furthermore, from NGS analysis of lysogens we propose that SGP-C genome might exist either as an episome, or both as integrated and temporary episome in the host cell and warrants further studies. Phylogenetic analysis revealed its similarity with *Salmonella* temperate phages belonging to family *Siphoviridae*. The encoded proteins by SGP-C genome have not showed homology with any known toxin and virulence factor. Although plenty of lytic bacteriophages against this pathogen are already reported, to our knowledge SGP-C is the first lysogenic phage against *S. gallinarum* reported so far.

## Introduction

*Salmonella enterica* serotype *gallinarum* biovar *gallinarum* (*S. gallinarum*) is an escalating threat to the modern poultry industry in developing countries (Kang et al., 2010; Alves Batista et al., 2018). The avian-adapted host- specific biovar *S. gallinarum* causes an acute systemic infection resulting in fowl typhoid with a high mortality rate from 80 to 100% (Hall et al., 1949; Penha Filho et al., 2016). Conventional antibiotic treatment can only partially control the diseases (Shivaprasad, 2000), however, it is challenging to completely confine its spread to unaffected birds. In the pre-antibiotic era, bacteriophages were first used as prophylactic agent against avian typhoid (*S. gallinarum*) (Summers, 2012), however, overuse of antimicrobials resulted in multi-drug resistant bacteria sparking a revival interest in exploring phages for their therapeutic potential (Brenner et al., 2020). Lytic bacteriophages are currently in use as therapeutic and prophylactic agent (Karthik et al., 2014; Wernicki et al., 2017) in poultry production. The temperate bacteriophages are usually studied for understanding their biology, role in bacterial virulence and antibiotic resistance, antibody display and vaccine vector, making recombinant phages and as a vehicle to transfer genetic material to their host.

Lysogenic or temperate bacteriophages either integrate their genome into the host chromosome or stably exist as an episome within the host cells (Clokie et al., 2011), and maintain either antagonistic or mutualistic association with the host (Fillol-Salom et al., 2019). They are naturally present in the environment as lysogens and can be targeted against bacteria that require special culturing conditions. Almost half of the bacteria sequenced so far are lysogens (Touchon et al., 2016). Temperate phages can transfer virulence and antibiotic resistance genes in bacterial populations (Penadés et al., 2015). These fitness genes are important for the bacterial survival. These phages have great impact on the evolutionary process of the bacterial species (Fortier and Sekulovic, 2013). Various phage encoded effector proteins are common among disease causing *Escherichia coli, Shigella* sp. and *Salmonella enterica* (Boyd, 2012).

Temperate bacteriophages can be used as a vehicle for genome editing of infectious microbes in environment due to their ability of integration into bacterial genome (Monteiro et al., 2019). Unique sequences; such as integrase and repressor genes of lysogen-lytic switch (Shin et al., 2014)of temperate phage are used extensively in bacteriophage recombineering and CRISPR/Cas9 genome editing (Monteiro et al., 2019). A recent study highlighted that imperfect matching of spacers with integrated temperate phage resulted in loss of CRISPR-Cas immunity in the host (Rollie et al., 2020). Temperate bacteriophages have ability to replicate in the lysogeny cycle as a prophage or produce virions in the lytic cycle (Domingo-Calap et al., 2016; Howard-Varona et al., 2017). These phages use CI-Cro bistable switch for conversion from lysogenic to lytic cycle (Schubert et al., 2007). In the present study, a temperate phage SGP-C was isolated against *S. gallinarum* and characterized. Virulence of temperate phage was estimated through various reported parameters. We further studied the phenomenon of superinfection immunity and spontaneous phage induction. In addition to whole genome analysis, transmission electron microscopy was done to reveal morphology of the virion particle.

## Materials and Methods

### Media, Chemicals and Reagents

For bacteriophage propagation, Typtic Soy Broth (TSB, Oxoid), Tryptic Soy Agar (1.5% agar), and Tryptic soy semi-solid agar (supplemented with 0.75% agar) were used. Polyethylene Glycol (PEG8000 10X, Sigma-Aldrich) and 1 M NaCl (Oxoid) were used for bacteriophage concentration. HCl (37%) and NaOH (1 M) was used for adjusting pH of the media. Glycerol (50%) was used for making bacteriophage glycerol stocks. Phage DNA was isolated through bacteriophage DNA isolation kit (Cat # 46850, NorgenBiotek), while lysogen DNA was isolated through DNA extraction kit (Cat # K0722, Thermo-scientific).

### Host Strain and Bacteriophage Isolation

Bacteriophage against host *S. gallinarum* (SG-C) was isolated from diseased chicken digesta obtained from local poultry facilities (Lahore, Pakistan) through the previously described method (Wang et al., 2020). Briefly, poultry digesta was mixed with autoclaved distilled water and incubated (30 min) for complete homogenization. The filtrate (0.45 μm syringe filtered) was incubated with SG-C for 24 h at 37°C in TSB broth followed by treatment with chloroform (1%) for 20 min. Broth was centrifuged at 10,000 × *g* for 10 min, and syringe filtered (0.45 μm) (Alvi et al., 2020a), followed by detection of phage through double-layer agar technique. Based on plaque size and morphology, phage was purified from a single plaque by re-propagation with the host strain (Bibi et al., 2016)for five rounds. The purified bacteriophages were syringe filtered (0.45 μm) and preserved in 25% glycerol at −80°C (Viti et al., 2000) for long-term storage and at 4°C for short-term storage.

### Determination of Host Range and Efficiency of Plating

In order to analyze host range of the temperate phage, spot assay (5 μl) was performed for initial screening followed by plaque assay to determine the efficiency of plating (EOP) against *S. gallinarum* (*n* = 15), *S. enteritidis* (*n* = 1), *S. typhimurium* (*n* = 1), *P. aeruginosa* (*n* = 3), *Klebsiella sp.* (*n* = 2), *Acinetobacter* (*n* = 2) and *S. aureus* (*n* = 2). EOP was calculated through the previously reported method (Khawaja et al., 2016) using SG-C as a reference strain. EOP values from 0.5 to 1 were classified as highly efficient, values between 0.2 to < 0.5 as moderately efficient and 0.0001 to < 0.2 as least efficient while < 0.0001 as in-efficient (Mirzaei and Nilsson, 2015; Esmael et al., 2021).

### Determination of SGP-C Stability

The pH stability of SGP-C was determined over a wide range of pH from 2 to 9. The specific pH of media was achieved using HCl (37%) and NaOH (1 M). One ml of phage lysate with known titer (1.1 × 10^10^ PFU/ml) was incubated in solutions with different pH range (2–9) at 37°C for 1 h. After incubation, the phage containing solution was neutralized to pH 7 with NaOH and HCl solution (Asif et al., 2020). The SGP-C thermal stability was determined by incubating 1 mL of phage lysate (1.1 × 10^10^ PFU/ml) at 4, 21, 37, 42, 50, 60, and 70°C for 1 h. Following incubation, bacteriophage titer was determined using double layer agar method (Alvi et al., 2020b). Difference in phage titer at different tested conditions, compared with original titer, was attributed to the effect of incubation temperature and pH (Rahmat Ullah et al., 2017). The SGP-C storage stability was determined after preserving the known titer of phage (1.1 × 10^10^PFU/ml) at −80 and 4°C for a period of 3 months followed by titer determination. Percentage reduction in phage titer was calculated through following formula:


Percentagetiterreduction=(Phageoriginaltiter-Phagetiteraftertreatment)×100/Phageoriginaltiter

### Development of SGP-C Putative Lysogens and Their Superinfection Immunity

TSA agar seeded with SG-C was spotted with SGP-C lysate with 10-fold dilution up to 10^–7^, followed by incubation at 37°C for 3 to 4 days (Harhala et al., 2018; Dedrick et al., 2019). After every 24 h, plates were observed for appearance of putative lysogen (mesas) within the plaque. The colonies from mesa were purified after three repeated rounds of purification and the isolates were further confirmed through patch test. Patch test was performed by two double layer TSA plates. On plate 1, SG-C was inoculated in the bottom layer and plate 2 without bacteria was labeled identically and was streaked with purified colonies from mesas; simultaneously, followed by incubation at 37°C for 24–48 h (Wottrich et al., 2020). Plates were observed for halo appearance around the streak line on plate 1 for induced phage activity (Wottrich et al., 2020). Log phase cultures of lysogens and *S. gallinarum* were used to make lawns for spot assay. Plates were spotted with 10-fold dilutions of SGP-C lysates followed by incubation at 37°C for 24 h to observe superinfection immunity due to prophage SGP-C (Kumar et al., 2008; Dedrick et al., 2019).

### Confirmation of SGP-C Lysogens Through PCR

The putative lysogens were confirmed through amplification of phage specific target. Whole genome of lysogens was extracted using bacterial genomic DNA extraction kit (cat#K0722) and used for PCR amplification of SGP-C tail length tape measure protein encoding gene (852 bp) inferred by bacteriophage genome sequence (Supplementary Table 1). The optimized amplification conditions were as follows: initial denaturation for 5 min at 95°C, followed by 35 cycles of denaturation at 95°C for 30 s, annealing at 60°C for 30 s, extension at 72°C for 30 s with final extension at 72°C for 7 min. SGP-C phage DNA and *S. gallinarum* genome were used as positive and negative control, respectively. Whole genome of lysogen was subjected to Illumina sequencing (Macrogen, Korea).

### Calculation of Burst Size and Latent Period

Burst size and latent period of SGP-C phage were determined by performing one-step growth curve according to previously described method (Owen et al., 2017). The SG-C strain was grown to exponential phase (1 × 10^9^CFU/ml) and incubated with SGP-C phage (1.1 × 10^8^ PFU/ml, MOI of 0.1) at 37°C for 5 min (Owen et al., 2017). The complete reaction mixture was centrifuged at 12,000 × *g* for 1 min. Supernatant was used for titer calculation, while pellet was re-suspended in 50 ml fresh autoclaved broth followed by incubation at 37°C for 1 h (Alvi et al., 2020b). Multiple samples were collected after every 5 min of incubation for titer calculation. Burst size was calculated by the formula:


Burst⁢Size=New⁢Phages⁢Released⁢(C)/Numberof⁢infecting⁢phages⁢(D).

Here C = B-A, where B is equal to phages after burst and A = total phages before burst. D = Total phages applied—free phages (Alvi et al., 2020b). Using the above method and calculations latent time and burst size of SGP-C phage were calculated.

### Quantification of SGPC Virulence

A standardized method based on three different parameters; virulence index (Vp), local virulence (Vi) and MV50, can be used to capture the bacteriophage infection dynamics (Storms et al., 2020). Briefly, SG-C was grown to log phase (1 × 10^8^ CFU/ml) and serial dilutions of phage SGP-C (1.1 × 10^9^) were prepared to achieve MOI from 10^–6^ to 10^0^. Equal volume (100 μl, 1 × 10^8^ CFU) of bacterial culture and each phage dilution were added to separate 96 wells plates in triplicates and optical density (OD_630*nm*_) was recorded at 30 min intervals for up-to 8 h until bacteria reached a stationary phase. The SG-C phage free culture was used as negative control. Reduction curves were generated and area under the OD_630*nm*_ versus time curve was calculated using trapezoid rule for each MOI and SG-C control (Storms et al., 2020; Townsend et al., 2020). The SGP-C phage local virulence (Vi), virulence index (Vp) and MV50 were calculated by using the formulas described in Supplementary Material.

### SGP-C Whole Genome Sequence and Phylogenetic Analysis

The SGP-C phage DNA was isolated through Phenol-Chloroform-Isoamyl alcohol (PCI) phage hunting protocol (Asif et al., 2020). Purified genome was subjected to Illumina sequencing (Macrogen, Korea). Genome assembly was then performed through SPAdes, the illumina reads trimmed through Trimmomatic using quality score of Phred + 33. Lifecycle of the phage was classified by online application PHACTS (McNair et al., 2012) and Phage AI^1^. PHASTER (Ríos-Sandoval et al., 2020) and GeneMarkS (Salisbury and Tsourkas, 2019) were used as the initial tools for open reading frames (ORFs) prediction. All predicted ORFs were confirmed and protein functions were assigned using RAST server and Blast results from NCBI against non-reductant protein database (McNair et al., 2018). Molecular weight and isoelectric point was calculated by Expasy^2^ while Pfam HMMER tool^3^ and HH phred^4^ were utilized for identifying the protein family based on its conserved domain matches. PHIRE tool was approached to reveal conserved regulatory elements in bacteriophage genome (Lavigne et al., 2004). The existence and location of rho-independent transcription terminators were determined using ARNold^5^ (Naville et al., 2011) combining RNAmotif and Erpin. Galaxy Phagepromoter^6^ tool was implied for promoters prediction. The ARAGRON program was used for searching putative tRNAs. Results were further confirmed through tRNA Scan-SE software^7^ (Chan and Lowe, 2019). For ruling out the presence of transmembrane helix and location of signal peptide cleavage sites in phage genome, TMHMM^8^ and SignalP-5.0 Server^9^ were used, respectively. The SMS explained codon usage frequency in the entire genome (Tabassum et al., 2018). Circular genomic map was constructed by Geneious Prime 2021.01. Protein sequences were analyzed under head-neck-tail module genes identification method in Virfam. It assigned viral genomes to morphotypes on the basis of sequence divergence of neck module, part of the head and tail proteins to previously characterized tailed viruses.

Putative protein sequence for tail-spike protein and DNA polymerase were selected for multiple sequence alignment done on ClustalW and further used for the construction of phylogenetic tree through UPGMA (unweighted pair group method with arithmetic mean) at Molecular Evolutionary Genetics Analysis Version X (MEGAX) using 500 bootstrap replicates. NCBI BLASTn for whole genome sequence was performed to determine the bacteriophage family of SGP-C (Kumar et al., 2016).

### Transmission Electron Microscopy

The morphology of bacteriophage SGP-C was determined through transmission electron microscopy performed at TEM services, Leicester University UK. The bacteriophage preparation for TEM analysis was done following a lab optimized protocol. Briefly, the filtered phage lysates were concentrated through centrifugal filter column (Amicon Ultra-4 centrifugal filter unit, Cat # C7719). The concentrated filtrate (10 μl) was applied to carbon coated grids, followed by addition of phosphor-tungstic acid (15 μl) before obtaining images.

## Results and Discussion

### SGP-C Produces Turbid Plaques

**FIGURE 1:**
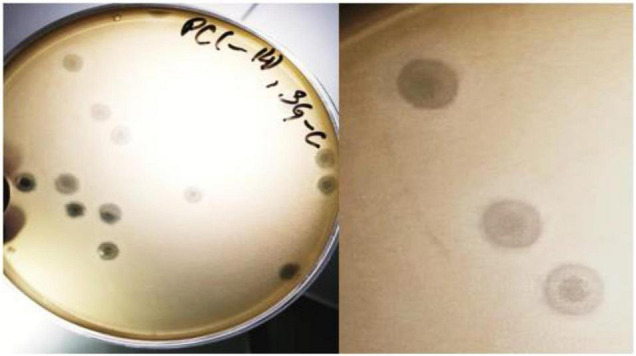
The SGP-C phage exhibiting bull’s eye morphology. Lysogen cells that are resistant to lysis, start growing inside the plaque thus giving it turbid appearance.

The SGP-C bacteriophage produced circular and turbid plaques having morphology of bull’s eye spot with 4–5 mm in diameter after 24 h of incubation (Figure 1). The turbid center of plaques became more visible after 4 days which agreed with the previously reported temperate phage, Che12 against *Mycobacterium tuberculosis*. Che12 phage formed turbid plaques after 5 days of incubation due to slow growing nature of bacteria (Kumar et al., 2008). The appearance of turbid plaque morphology can be due to the emergence of lysogens inside a plaque that do not undergo lysis and appeared as bull’s eye spot. Appearance of a cloudy center in plaque could be taken in account for the formation of lysogens (Shin et al., 2014; Staes, 2019).

### The SGP-C Phage Demonstrated 100% Infectivity Against *S. gallinarum* Isolates

The SGP-C showed activity against all the tested strains of *S. gallinarum* (*n* = 15) in spot as well as plaque assay. However, it showed lysis of tested S. *typhimurium* and *S. enteritidis* only on spot assay which may be due to the phenomena of “lysis from without.” The bacteriophage produced higher EOP values against SG-F, SG-36, SG-47, SG-60 and SG-18, while moderate EOP values were recorded against rest of the tested *S. gallinarum* isolates (Supplementary Table 2). A previous study reported a broad host range lytic phage SAL-PG against *S. gallinarum* and *S. pullorum* with 77.77% infectivity (Rahaman et al., 2014). In another study phage LPST153 was active against 5 tested strains of *S. gallinarum*, but showed lower EOP values (Islam et al., 2020). The SGP-C was found spot negative against all tested strains of *Pseudomonas aeruginosa*, *Klebsiella* sp, *Acinetobacter*, and *S. aureus*, which agreed with the previously reported lytic phage LPST153 (Islam et al., 2020).

### The SGP-C Showed Stability at Broad Physiological pH and Temperature Range

**FIGURE 2:**
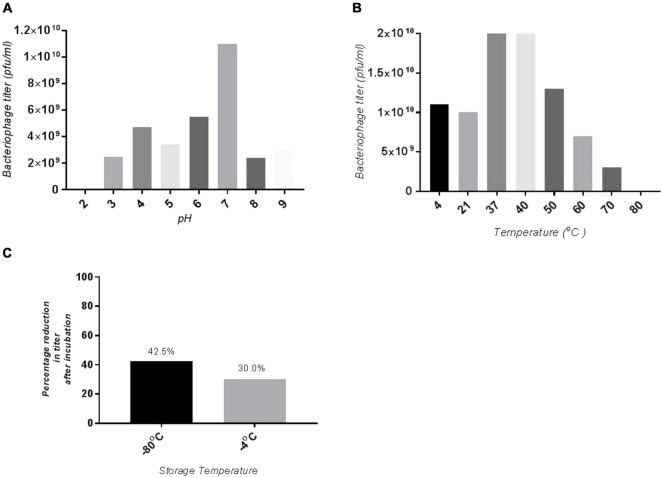
Stability of SGP-C at variety of physiological parameters **(A)** optimum pH for phage stability is pH 7 hence giving maximum phage titer, whereas least stability seen at pH 2, **(B)** maximum phage stability observed at 37 and 42°C which decrease with increase in temperature, **(C)** SGP-C phage is more stable at 4°C as compared with –80°C for 3 months.

The SGP-C phage remained viable at pH range 4.0–9.0, which agreed with previously reported phage LPST153 that showed stability at pH range of 4.0–9.0, (Islam et al., 2020). SGP-C showed maximum stability at pH 7.0 and least stable at pH 2.0 (Figure 2A). The optimum activity of SGP-C was exhibited at temperature range of 37–42°C which attributed to *S. gallinarum* ability to grow at 42°C. The phage remained stable at 4, 21, 37, and 42°C (Figure 2B); however, after 42°C, phage viability was reduced significantly, which is similar to the previously reported stability pattern of lytic phages against *S. gallinarum* (Hong et al., 2013). The shelf life of SGP-C was higher at 4°C than at −80°C (Figure 2C).

### SGP-C Phage Lysogens Exhibited Superinfection Immunity

**FIGURE 3:**
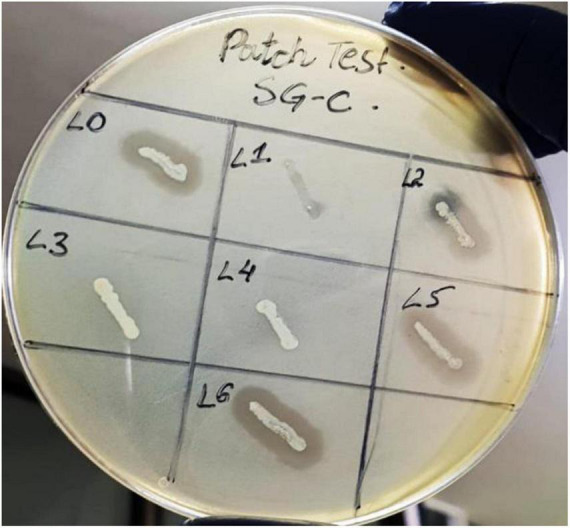
Patch test for SGP-C phage lysogens SGPC-L0, SGPC-L2, SGPC-L5 and SGPC-L6 over SG-C showed clearance around streak line due to spontaneously induced SGP-C phage activity.

The patch test is a reliable method that can be used for rapid screening of lysogens (Cumby et al., 2012). We isolated pure colonies of lysogens to determine the acquired resistance against super infection by the same phage. Among seven purified lysogens four were positive for the patch test thus called as SGP-C lysogens SGPC-L0, SGPC-L2, SGPC-L5 and SGPC-L6 (Figure 3). All lysogens were non-permissive to SGP-C phage lysate when assessed by spot assay and double agar overlay method contrary to the SG-C control, indicating the superinfection immunity against same phage. This phenomenon has been previously studied for Salmonella phage P22 and Salmonella phage L (Mahony et al., 2008). SGP-C might be giving super infection immunity through mechanism similar in other reported phages or using some novel mechanism that need further research. In non-lysogenic colonies, phage resistance might be prevented due to down regulation of phage receptors genes (Oechslin, 2018).

### Lysogen Confirmation Through PCR

**FIGURE 4:**
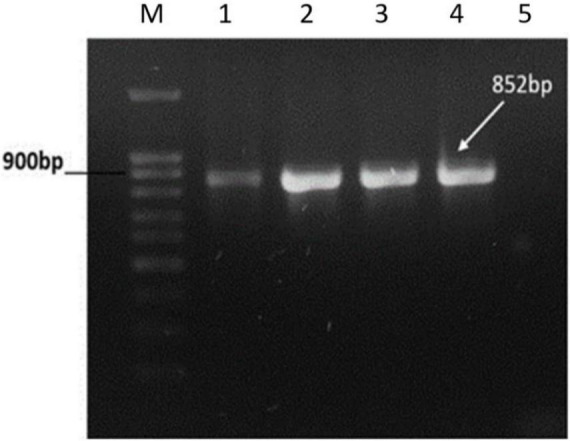
PCR amplification of Tail Length tape Measure protein gene of SGP-C phage from genomic DNA of lysogens. PCR amplified target band of 852bp from SGP-C positive control (Lane 1), SGP-C Lysogens (Lane 2–4), while no product was amplified in SG-C negative control (lane 5). Lane M with 100 bp plus DNA ladder (Cat # 304150 Bioron).

To confirm the presence of prophage genome into the lysogens, the tail length tape measure protein (TLTMP) encoding gene was amplified for detection of SGP-C phage using genomic DNA of lysogen. Amplification of a ∼0.8 kb fragment using the TLTMP encoding gene specific primers, indicate the presence of SGP-C phage in the *S. gallinarum* genome. The tested lysogens of *S. gallinarum SGPC-L5* and *SGPC-L6* confirmed the presence of SGP-C phage DNA in the lysogens (Figure 4). Whole genome sequence of lysogen SGPC-L5 was done and read mappings revealed areas of increased coverage, especially over prophage regions (Deutsch et al., 2018). It suggests that SGP-C might exist either as episomal (P1 type), or as integrated and episomal phage genome in host cell (Łobocka et al., 2004), which needs further studies.

### SGP-C Exhibit Extended Latent Period

**FIGURE 5:**
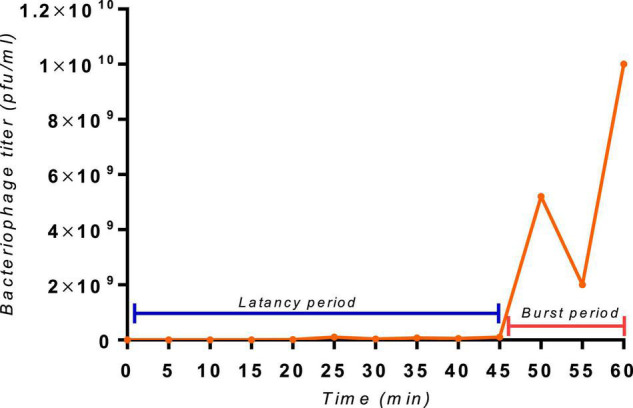
The one-step growth curve of the SGP-C phage, with an indication of extended latent period of 45 min and a second burst at 60 min with a burst size of 187 virions per cell.

Bacteriophage burst size and latency period are two important parameters of its infection biology (Alvi et al., 2020b). The SGP-C phage showed relatively extended latent period of 45 min (Figure 5) with burst size of 187 virions per infected cell presenting reductive infection (Dennehy and Abedon, 2020). However, another burst can be observed around 60 min with a rise in viral titer. In lysogeny multiple bursts can be observed as a part of infection lineage (Dennehy and Abedon, 2020)or as a consequences of lysogen binary fission and prophage induction. Reductive infection can last indefinitely and have relatively longer latent period (Dennehy and Abedon, 2020). Previously reported temperate phage SPN9CC of *Salmonella* Sp. exhibited longer latency period (30 min) and smaller burst size (220) (Shin et al., 2014).

### SGP-C Is Moderately Virulent Phage

**FIGURE 6:**
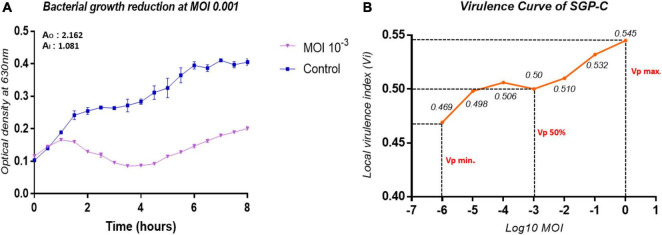
The virulence kinetics of SGP-C, **(A)** SG-C Bacterial growth reduction due to SGP-C at MOI 0.001, **(B)** SGP-C phage virulence curve generated using local virulence (y-axis) at respective MOI (x-axis) indicating MV50 = 0.001.

The infection dynamics of SGP-C were further determined by estimating its local virulence (vi) at given MOI upon infection to SG-C strain at 37°C for up-to 8 h. The SGP-C exhibited maximum local virulence V*i* = 0.545 at MOI 1 (Figure 6A). Earlier the *vi* approaches to 1 on the virulence curve from lower to higher MOI, more virulent the phage is (Storms et al., 2020). Previously, T5 phage of *Escherichia coli* was found least virulent, exhibited measurable V*i* values at MOI ≥ 0.01 and V*p* = 0.17 (Storms et al., 2020). In the present study SGP-C virulence index (V*p*) was 0.5 and MV50 = 0.001 (Figure 6B). These findings suggested that SGP-C phage is moderately virulent.

### SGP-C Genome Size Is 42 Kbp With Intact Prophage Region

High throughput sequencing reads of SGP-C built up a continuous genome sequence of 42,362bp exhibiting 50.04% GC content, which is quite comparable to φSG-JL2 phage of *S. enterica* with 50.9% GC content (Kwon et al., 2008). Restriction endonuclease mapping with *Hin*dIII enzyme (Supplementary Figure 1) confirmed that the genome exist in circular form in the purified phage particles (Bao et al., 2011; Akhwale et al., 2019). The Whole genome megaBlast analysis of SGP-C phage having intact prophage region revealed that Salmonella phage SETP3 intact genome (Accession no. EF177456.2) exhibits highest query coverage (84%) with similarity index of 93.85%. A total of 63 ORFs were predicted in SGPC phage genome (Supplementary Table 3).

**FIGURE 7:**
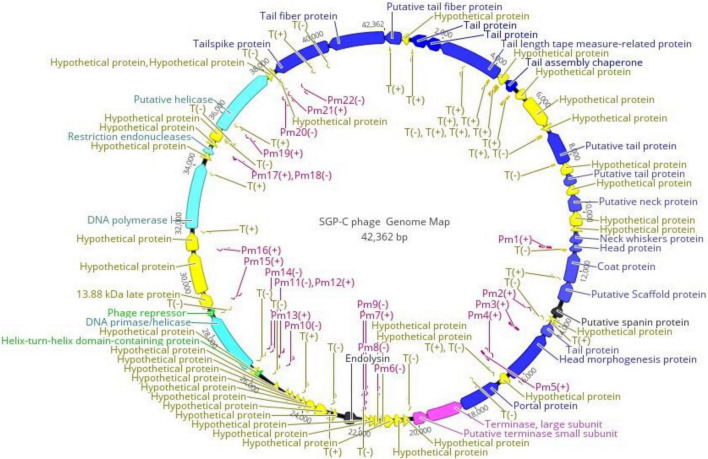
Functional Genome map of SGP-C, generated through Geneious Prime 2021.01. Functions were assigned on basis of database homology to Blastp and HHphred. Arrow head is directed in the direction of ORF. Color code is as: yellow for hypothetical proteins, blue for structural proteins, purple and light blue for DNA packaging and metabolism proteins, green for lysogenic module and black for bacterial lysis. Pm refers to the promoters and T refers to Phage terminators while ± indicates its presence on DNA strand.

PHACTS algorithm confidently predicted life-cycle of phage as temperate with average probability of 0.557. Among predicted ORFs, 24 (38.09% of the genome) were assigned functions through annotation, while other 39 ORFs (61.90% of the genome) lacked any data base matches and named as “hypothetical proteins.” The shortest ORF61 containing 93 bp encoded for hypothetical protein while the largest ORF59 with 2,466 bp encoded for a putative helicase. The isoelectric point (pI) of predicted proteins was between 4.0 and 10. Among all functional proteins, helix-turn-helix domain-containing protein encoded by ORF46 was the smallest sized protein of 6.6 kDa with pI of 9.65, while ORF59 encoded for largest protein of ∼92.5 kDa with pI 8.80. Conserved phage-specific putative regulatory element with related sequence of TAATAGTACCCTATTATGTT were detected in 28 genes. A total of 22 phage promoters and 36 rho-independent transcription terminators were found in the phage genome (Supplementary Tables 4, 5). Signal protein cleavage site was observed in protein product of ORF8 and 41 with the cleavage position between 18 to 19 and 19 to 20 amino acid, respectively. Three transmembrane helixes were found in the genome among ORF31, ORF43 (hypothetical proteins) and ORF62 (tail-spike protein). Most frequently (26.77/100) occurring codon was CGG encoding for arginine. The fact about lysogenic phages harboring less tRNAs is supported by SGP-C (0 tRNA). Absence of tRNA questioned phage fitness through lowering the rate of protein synthesis (Bailly-Bechet et al., 2007). Annotated proteins were categorized as structural proteins, DNA metabolism and packaging proteins, lysogenic module and bacterial lysis proteins (Figure 7). The SGP-C did not encode for known *Salmonella* virulence, antibiotic resistance and toxin proteins as reported for SRϕ1.

Like tailed phages, structural proteins in SGPC also followed modular organization and clustered together on negative strand. Nevertheless, Virfam revealed that SGP-C exhibit two classes from the head (major capsid protein, terminase at ORF 20 and 28, respectively), five from the neck (Portal, Ad1, Hc1, Tc1, Ne1 at ORF 27, 16, 14, 12, and 13, respectively) and one from the tail (major tail protein at ORF 11) (Supplementary Figure 2). Together these head-neck-tail gene shows order of Siphoviridae Type1 (Cluster 5). Where in the genome, capsid proteins including ORF 19, ORF 20, ORF 21, and ORF 25 present downstream of portal protein and those encoding for terminase sub-units. Head to tail joining neck proteins (ORF 15 and ORF 18) were encoded between genes translating major capsid and 7 tail assembly proteins (ORF 1, ORF 3, ORF 4, ORF 5, ORF 7, ORF 11, ORF 13, ORF 62, and ORF 63). Tail assembly proteins were present at the end of Structural module. These 15 structural proteins thus arrange in long tailed icosahedral symmetry similar to members of Siphoviridae family. Upstream of the Capsid proteins, DNA packaging module including portal protein (ORF 27) and terminase complex were encoded (ORF 28 and ORF 29). Like most of the tailed phages heterodimer terminase with large catalytic component with ATPase and endonuclease activities (ORF 28) and a small sub-unit (ORF29) for DNA-recognition were present (Rao and Feiss, 2015). A single phage promoter Pm6 (−) on negative strand upstream of Terminase complex control the translational process of DNA packaging and Structural module (Figure 7).

Bioinformatics approaches allowed identification of genes encoding for proteins involved in DNA metabolism that were not clustered together on genome. SGPC phage DNA was observed to be semiautonomous and likely to borrow many of the host replisome proteins for metabolism (Kaliniene et al., 2021). ORF 53 for DNA polymerase I (DNA_pol_A domain), Putative helicases at ORF 59 having Hom_end-associated Hint domain, restriction endonucleases at ORF 56 and ORF 58 having VRR_NUC and HNH_3 domains, respectively were not found co-localized on the genome. Recombination associated protein of family DUF2800 encoded by ORF 51, has been predicted by Virfam to belong to the Rad52 family. It included the DNA single-strand annealing proteins related to Rad52, such as RecT, Red-beta and ERF that function in RecA-dependent as well as RecA-independent DNA recombination pathways. Notably, according to the SCOP family, another ORF 48 apart from domain RecA protein-like (ATPase domain), specific domains of Rad51 or helicases was identified (Kaliniene et al., 2021).

SGP-C exhibited two central components of lysogeny module with 2.4 bp region apart. These components commit for the outcome of “lysis versus lysogeny” decision: λ repressor cI gene for lysogeny and a bistable genetic switch Cro gene for lytic growth. ORF49 related to λ repressor Cro is a DNA-binding transcriptional regulator with Helix-turn-Helix domain (HHphred probability 97.73%, E value 0.00008). Multifunctional ORF 46, a transcriptional regulatory element, might functions as cI with Helix-turn-Helix_17 domain (HHphred probability 98.37%, E value 0.0000013) and Phage_Cox (HHphred probability 98.38%, E value 0.0000021). Phage_Cox might perform excision during lytic cycle of SGP-C (Yu et al., 2020). A key phenomenon of Cro gene downregulation/Genes silencing guided by affinity-based binding of phage repressor proteins, thus decide fate of temperate phage genetic diversion (Aggarwal et al., 1988; Waldor and Friedman, 2005; Hobbs and Abedon, 2016). The repressor protein has long been manipulated to study lysogeny in bacteriophage recombineering (Zhang et al., 2013; Monteiro et al., 2019).

For lysogenic to lytic switching, phage lytic protein (ORF37) was denoted as endolysin. However, no holin protein was found in SGP-C genome (Zhao et al., 2019). Third functional class of lysis proteins spanin (ORF22) was found in SGP-C genome, which play a role in disrupting the bacterial outer membrane or plasma membrane during final step of host lysis (Matamp and Bhat, 2019). In most tailed phages lysis genes are in close proximity to make lysis cassette while in SGP-C lytic proteins were found 8.7 Kbp apart (Fernandes and São-José, 2018).

**FIGURE 8:**
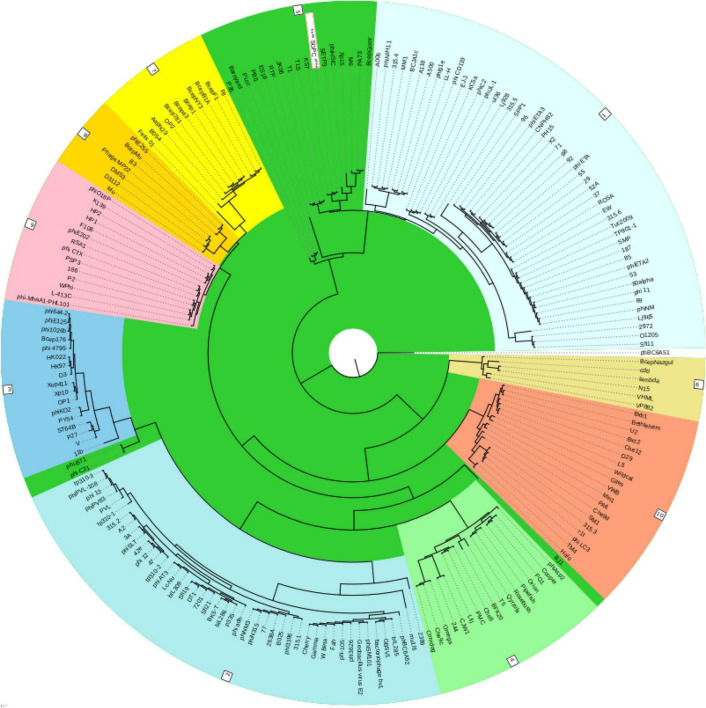
Classification of the SGPC Phage along other related Phages of siphoviridae family in ACLAME database (Leplae et al., 2009) generated by Virfam analysis (SGPC Phage in text box with red border and white background).

**FIGURE 9:**
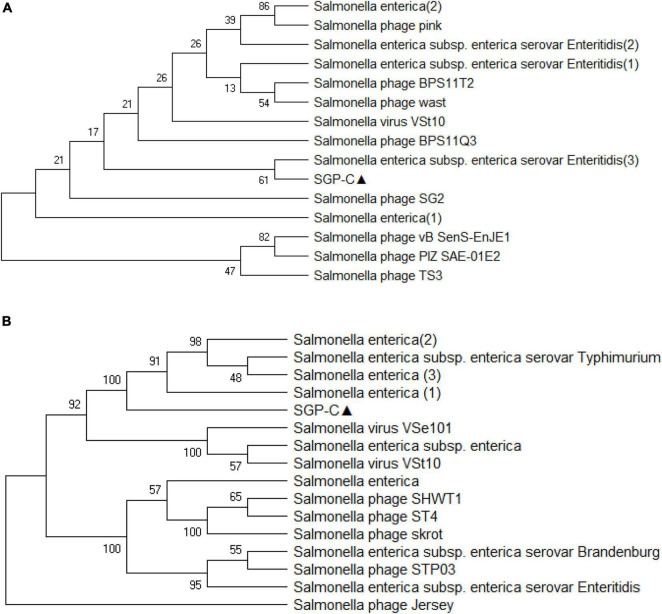
The evolutionary history was conducted using the UPGMA method in MEGA.X based on top 15 and 16 protein homologs found on GenBank search for Tail-spike protein **(A)** and DNA polymerase **(B)**. Total of 500 bootstrap replicates in which tree associated taxa clustered together are shown next to branches. Poisson correction method along with units of the number of amino acid substitute per site are used for determining evolutionary distances. There was a total of 684 positions in the first and 774 in second data set.

Virfam analysis grouped SGP-C along with Salmonella phage SETP3 intact genome (Accession no. EF177456.2) of *Siphoviridae* family but with linear genome (Figure 8). Phylogenetic analysis of phage Tail spike protein and DNA polymerase revealed significant clustering of phage with proteins of similar domains form other phages as well as Salmonella species (Figure 9). Phylogenetic tree based on Tail spike protein showed evolutionary relation among SGP-C, Salmonella phage SG2, BPS11Q3 while DNA polymerase indicates that SGP-C is distantly related *Salmonella* virus VSe101 and VSt10 (Figure 9). NCBI nucleotide and protein BLAST sequence homology grouped SGP-C bacteriophage among the members of family *Siphoviridae* similar to the majority of the *Salmonella* temperate phages (e.g., FSL SP-088, FSL SP-099, FSL SP-039) belonging to the same family (Switt et al., 2013).

The annotated whole-genome sequence of bacteriophage SGP-C was submitted to the NCBI GenBank database (accession no. OK169616).

### *Siphoviridae* Morphotype of SGP-C

**FIGURE 10:**
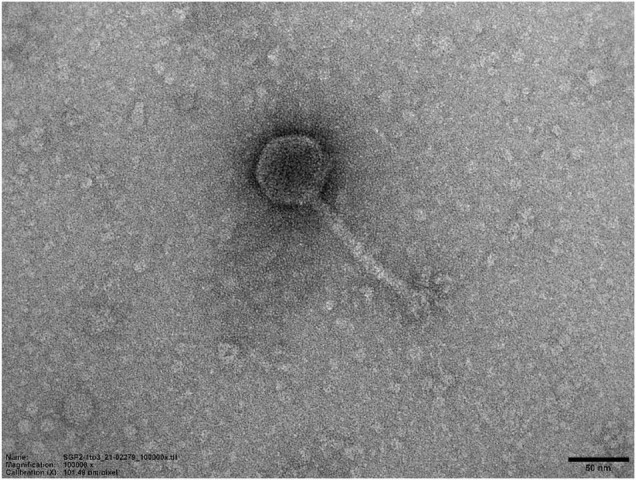
Transmission electron micrograph of SGP-C (50 nm), demonstrating long flexible non-contractile tail with an icosahedral head morphotype.

Transmission electron microscopy demonstrated that SGP-C phage possessed an icosahedral head with a diameter of 59.77 nm and tail length and width of approximately 111.47 and 9.104 nm, respectively (Figure 10). On the basis of current International Committee on Taxonomy of Viruses classification system, SGP-C is classified as member of family *Siphoviridae* family, along with other *Salmonella* phages like LPST10 and vB_SenS-Ent1 (Turner et al., 2012; Huang et al., 2018).

## Conclusion

In this study, SGP-C, a temperate phage against *S. gallinarum* was isolated from diseased poultry digesta and was found active against all the tested *S. gallinarum* strains, with stability at different temperature and pH. The SGP-C phage is moderately virulent with extended latent time. The SGP-C being devoid of any known toxic or virulence gene can be useful for recombineering of the host and to control *S. gallinarum* through loading it with gene specific nucleases like CRISPR/Cas9 in future studies.

## Data Availability Statement

The datasets presented in this study can be found in online repositories. The names of the repository/repositories and accession number(s) can be found below: GenBank BankIt2498468SGPC, accession no: OK169616.

## Author Contributions

SI and HafT equally contributed as first authors in the experimentation, analysis, and writing of the manuscript. HafT, HamT, and ZH helped in performing additional experiments and analysis during the revision of the manuscript. AB and SR contributed to designing research and proofreading the manuscript.

## Author Disclaimer

The views expressed herein do not necessarily represent those of IDRC or its Board of Governors.

## Conflict of Interest

The authors declare that the research was conducted in the absence of any commercial or financial relationships that could be construed as a potential conflict of interest.

## Publisher’s Note

All claims expressed in this article are solely those of the authors and do not necessarily represent those of their affiliated organizations, or those of the publisher, the editors and the reviewers. Any product that may be evaluated in this article, or claim that may be made by its manufacturer, is not guaranteed or endorsed by the publisher.

## Footnotes

1
www.phage.ai

2
https://web.expasy.org/protparam/

3
http://www.ebi.ac.uk/Tools/hmmer/

4
https://toolkit.tuebingen.mpg.de/

5
http://rssf.i2bc.paris-saclay.fr/toolbox/arnold/index.php#Results

6
https://galaxy.bio.di.uminho.pt/

7
http://lowelab.ucsc.edu/tRNAscan-SE/

8
http://www.cbs.dtu.dk/services/TMHMM/

9
http://www.cbs.dtu.dk/services/SignalP/
