# Supplementary material for: SGP-C: A Broad Host Range Temperate Bacteriophage; Against Salmonella gallinarum
Source: Front Microbiol. 2022 Jan 12;12:768931. doi: 10.3389/fmicb.2021.768931 (PMC8790156; doi:10.3389/fmicb.2021.768931)
Supplement: Supplementary file 1 [file Data_Sheet_1.pdf]

SGP-C Infection dynamics

MV50 is the MOI at which 50% of the bacterial population is cleared by phage.

Local Virulence (Vi): Local virulence can be calculated by using the following formula:

Vi=1-(Ai/Ao)

V*i* can be measured at the scale of 0 to 1, where 0 indicates the complete absence and 1 represents the maximum theoretical virulence.

Here Ai is the area under curve at specific MOI of SGP-C, while Ao is the area under the curve for phage free bacterial control SG-C. At MOI 0.01 the local virulence can be calculated as follows:

$V_{0.01}$= 1-(1.081/2.162)

=0.50

Then using the values of local virulence at different MOI virulence curve were generated between MOI (x-axis) and respective local virulence (y-axis).

Virulence curve used for calculating virulence index by using following formula:

Vp= Ap/Amax

For calculating Vp, MOI were converted to log10 (MOI) and were plotted against the local virulence (Fig 8). From curve (Ap) was calculated by using trapezoid method. Ap is the area covered by SGP-C phage in experiment while Amax is the area for maximum theoretical virulence.

$V_{tB\text{-}SGP\text{-}C}$ = $A_{SGP\text{-}C}$ /Amax

=3.053/6

=0.508

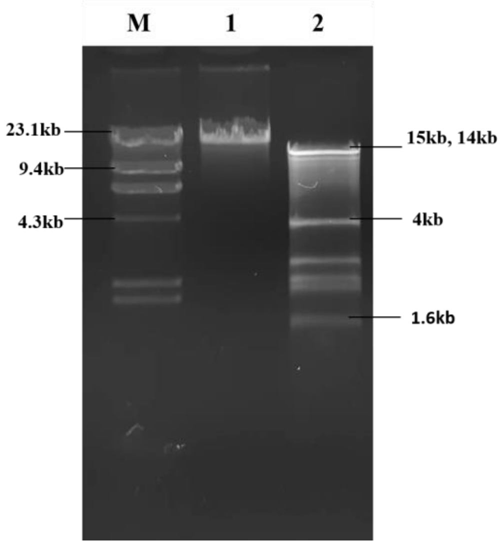

Figure S1: SGP-C phage genome restriction digestion with HindIII enzyme. Lane M contains Phage Lambda DNA ladder, Lane 1 with uncut SGP-C DNA and Lane 2 restricted with Hind3 enzyme.

Table S1: Primer pair for Tail Length Tape Measure Protein (2310bp) of SGP-C phage.

**Primer**	**Sequence**	**Tm**	**Primer Binding site**	**Product size**
FP-TLTP	CGTTGGCGTCAATATGCTGG	60.0	709bp	852bp
RP-TLTP	TCTGAGCGACATACGCATCC	60.0	1560bp	

Table S2: SGP-C phage Efficiency of Plating (EOP)

**Bacterial Host**	**SGP-C titer with SG strains**	**EOP**
SG-C (original host)	1.1x 10^{10}	1.00
Sg-4	3.3x 10^{9}	0.3
Sg-A	4.5x 10^{9}	0.41
Sg-B	4.5x 10^{9}	0.41
Sg-D	5.0x 10^{9}	0.45
Sg-E	4.0x 10^{9}	0.36
Sg-F	6.0x 10^{9}	0.55
Sg-G	5.0x 10^{9}	0.45
Sg-18	6.0x 10^{9}	0.55
Sg-12	5.0x 10^{9}	0.45
Sg-36	6.7x 10^{9}	0.61
Sg-47	6.0x 10^{9}	0.55
Sg-67	5.0x 10^{9}	0.45
Sg-60	6.0x 10^{9}	0.55
Sg-29	4.5x 10^{9}	0.45

Table S3: The comprehensive characterization of SGP-C genome and characteristics of the annotated proteins. Putative proteins with known function are highlighted

Gene #	Location on genome		Total bp	CDS	Total GC content	Function	Protein family	Total aa	Similar protein	Similarity index	Accession no.	Mol. Wt (kDa)	TMH	Isoelectric point (pH
	Start	stop												
1.	607	2	606	-	51	Putative tail fiber protein	Bacteriophage lambda tail assembly protein I	202	Salmonella phage SETP3	99.50%	YP_001110802.1	22.0	0	5.02
2.	861	598	264	-	52	Hypothetical protein	DUF1460 ; Protein of unknown function (DUF1460)	87	Salmonella phage SETP13	100%	YP_008767029.1	9.8	0	6.34
3.	1475	960	516	-	50	Tail protein	Phage_tail_L ; Phage minor tail protein L	171	Salmonella phage SE2	98.83%	YP_005098154.1	19.2	0	4.20
4.	1972	1472	501	-	51	Tail protein	Phage_min_tail ; Phage minor tail protein	166	Salmonella phage SS3e	99.40%	YP_308655.1	18.7	0	4.67
5.	4283	1974	2310	-	52	Tail length tape measure-related protein	TMP_2 ; Prophage tail length tape measure protein	769	Salmonella phage pink	98.57	QIN98000.1	81.6	0	4.97
6.	4611	4276	336	-	46	Hypothetical protein	DUF1799 ; Phage related hypothetical protein (DUF1799)	111	Salmonella phage SETP3	95.50%	YP_001110852.1	12.6	0	5.24
7.	5003	4638	366	-	51	Tail assembly chaperone	Phage_TAC_4 ; Phage tail assembly chaperone	121	Salmonella phage SETP3	97.52%	YP_001110851.1	13.7	0	4.76
8.	5232	5561	330	+	44	Hypothetical protein		109	Salmonella phage STP03	89.91%	APM00265.1	11.6	0	4.97
9.	5624	6760	1137	+	50	Hypothetical protein		378	Salmonella phage SETP13	92.33%	YP_008767022.1	42.6	0	9.29

10.	6757	6987	231	+	43	Hypothetical protein	DUF3310 ; Protein of unknwon function (DUF3310)	76	Salmonella phage STP03	97.37	APM00267.1	8.9	0	5.08
11.	8185	7016	1170	-	51	Putative tail protein	Phage_tube_2 ; Phage tail tube protein	389	Salmonella phage pink	97.43%	QIN97994.1	41.1	0	4.29
12.	8604	8185	420	-	47	Hypothetical protein	DUF4128 ; Bacteriophage related domain of unknown function	139	Salmonella phage Jersey	94.96%	YP_008239729.1	15.1	0	4.23
13.	8999	8604	396	-	46	Putative tail protein	HK97-gp10_like ; Bacteriophage HK97-gp10, putative tail-component	131	Salmonella phage SETP13	100%	YP_008767017.1	14.5	0	10.48
14.	9355	8996	360	-	49	Hypothetical protein	Phage_attach ; Phage Head-Tail Attachment	119	Salmonella phage SETP13	98.32%	YP_008767016.1	13.1	0	10.13
15.	9960	9355	606	-	53	Putative neck protein		201	Salmonella phage SETP7	95.52%	YP_008767187.1	20.6	0	9.58
16.	10472	9963	510	-	51	Hypothetical protein	DUF3199 ; Protein of unknown function (DUF3199)	169	Salmonella phage vB_SpuS_Sp4	97.63%	AWY03048.1	17.8	0	4.45
17.	10664	10476	189	-	50	Hypothetical protein		62	Salmonella phage FSL SP-101	98.39%	YP_009617930.1	7.1	0	4.61
18.	11051	10701	351	-	48	Neck whiskers protein		116	Salmonella phage S101	98.28%	AXC39560.1	12.2	0	4.46
19.	11347	11063	285	-	55	Head protein	Phage_head_fibr ; Head fiber protein	94	Salmonella phage vB_SenS_PVP-SE2	97.87%	AST15498.1	9.3	0	10.48

20.	12457	11408	1050	-	54	Coat protein	Gp13-like ; Major capsid protein 13-like	349	Salmonella phage FSL SP-101	99.14%	YP_009617927.1	37.8	0	4.37
21.	13162	12461	702	-	50	Putative Scaffold protein		233	Salmonella phage SETP3	99.14%	YP_001110836.1	25.7	0	6.18
22.	13742	13356	378	-	53	Putative spanin protein		128	Salmonella phage SETP7	96.03%	YP_008767179.1	14.2	0	8.43
23.	13851	14009	159	+	47	hypothetical protein		52				6.0	0	9.94
24.	14519	14061	459	-	49	Tail protein		152	Salmonella phage wksl3	97.37%	YP_009608684.1	16.4	0	4.41
25.	16381	14522	1860	-	52	Head morphogenesis protein	Phage_min_cap2 ; Phage minor capsid protein 2	619	Salmonella phage demigod	97.09%	QIN98040.1	67.4	0	4.99
26.	16484	16852	369	+	45	Hypothetical protein		122	Salmonella phage SETP13	100%	YP_008767003.1	14.2	0	4.34
27.	18357	16888	1470	-	54	Portal protein	Phage_prot_Gp6 ; Phage portal protein, SPP1 Gp6-like	489	Salmonella phage pink	98.57%	QIN97976.1	53.9	0	4.48
28.	19641	18370	1272	-	51	Terminase, large subunit	Terminase_1 ; Phage Terminase	423	Salmonella phage STP03	99.53%	APM00285.1	47.6	0	6.30
29.	20137	19631	507	-	54	Putative terminase small subunit	Phage_term_sma ; Putative bacteriophage terminase small subunit	168	Salmonella phage vB_SenS-EnJE1	100%	QGJ84413.1	18.9	0	6.29
30.	20501	20271	231	-	44	Hypothetical protein		76	Salmonella phage Ent1	97.37%	YP_007010511.1	8.2	0	6.23
31.	20812	20513	300	-	45	Hypothetical protein		99	Salmonella phage Ent1	98.99%	YP_007010510.1	11.7	1	10.25
32.	21165	20809	357	-	49	Hypothetical protein	DUF2774 ; Protein of unknown function (DUF2774)	118	Salmonella phage SETP13	99.15%	YP_008767066.1	13.6	0	8.24
33.	21317	21162	156	-	50	Hypothetical protein	DUF2737 ; Protein of unknown	51	Salmonella phage LSPA1	92.16%	YP_009113202.1	6.0	0	11.43

							function (DUF2737)							
34.	21511	21314	198	-	42	Hypothetical protein	DUF6501 ; Family of unknown function (DUF6501)	65	Escherichia phage vB_EcoS_XY1	84.62%	QIG59228.1	7.2	0	4.05
35.	21693	21508	186	-	47	Hypothetical protein		61	Salmonella phage SETP7	95.08%	APM00294.1	6.8	0	8.47
36.	21893	21699	195	-	45	Hypothetical protein		64	Salmonella phage SETP13	96.88%	YP_008767062.1	7.0	0	4.52
37.	22567	22079	489	-	55	Endolysin	Phage_lysozyme ; Phage lysozyme	162	Salmonella phage vB_SenS_phi135	97.53%	AYP69682.1	17.3	0	10.35
38.	22989	22837	153	-	45	Hypothetical protein	DUF6107 ; Family of unknown function (DUF6107)	50	Salmonella phage SF4	100%	QDH44753.1	5.7	0	4.59
39.	23632	23198	435	-	48	Hypothetical protein		144	Salmonella phage SE2	97.92%	YP_005098116.1	15.5	0	4.36
40.	24003	23638	366	-	52	Hypothetical protein		121	Salmonella phage vB_SpuS_Sp4	99.17%	AWY03010.1	13.8	0	11.18
41.	24209	24006	204	-	53	Hypothetical protein		67	Salmonella phage Shelanagig	100%	QEG07391.1	7.5	0	7.42
42.	24445	24206	240	-	54	Hypothetical protein		79	Salmonella phage vB_SenS_phi135	65.56%	AYP69680.1	8.9	0	5.72
43.	24664	24458	207	-	47	Hypothetical protein		68	Salmonella phage SETP13	95.16%	YP_008767053.1	8.1	1	10.44
44.	24846	24661	186	-	48	Hypothetical protein	DUF2680 ; Protein of unknown function (DUF2680)	61	Salmonella phage skrot	96.72%	QIO03728.1	7.0	0	4.46

45.	25310	25107	204	-	46	Hypothetical protein		67	Salmonella phage vB_SenS_phi135	96.97%	AYP69705.1	7.4	0	9.53
46.	25948	26118	171	+	52	Helix-turn-helix domain-containing protein	Phage_Cox ; Regulatory phage protein cox ; HTH_17	56	Salmonella phage Shelanagig	100%	QEG07385.1	6.6	0	9.65
47.	26115	26348	234	+	43	Hypothetical protein	DUF4423 ; Domain of unknown function (DUF4423)	77	Salmonella phage SETP7	100%	YP_008767217.1	8.6	0	9.55
48.	26405	28591	2187	+	54	DNA primase/helicase	Rad51 ; Rad51	728	Salmonella phage vB_StyS-sam	99.45%	BBO66012.1	80.5	0	4.82
49.	28824	28606	219	-	53	Phage repressor Helix-turn-helix domain	P22 –Cro;DNA-binding transcriptional regulator ; Cro	72	Salmonella phage SETP13	97.18%	YP_008767044.1	78.9	0	9.51
50.	28959	29495	537	+	45	13.88 kDa late protein	DUF4363 ; Domain of unknown function (DUF4363)	178	Salmonella phage NBSal007	89.56%	QMP81625.1	20.1	0	4.27
51.	29537	30967	1431	+	50	Hypothetical protein	DUF2800 ; Protein of unknown function (DUF2800), Rad52-like	476	Salmonella phage BPS11Q3	95.80%	YP_009322843.1	53.2	0	6.53
52.	31049	31672	624	+	48	Hypothetical protein	DUF2815 ; Protein of unknown function (DUF2815)	207	Salmonella phage SETP7	100%	YP_008767210.1	23.4	0	4.68
53.	31829	34132	2304	+	51	DNA polymerase I	DNA_pol_A ; DNA polymerase family A	767	Salmonella phage STP03	87.96%	APM00319.1	86.8	0	7.87

54.	34122	34253	132	+	47	Hypothetical protein		43	Salmonella phage BPS11Q3	86.05%	YP_009322846.1	4.8	0	7.35
55.	34336	34530	195	+	48	Hypothetical protein		64	Salmonella phage FSL SP-101	96.88%	YP_009617954.1	7.3	0	4.11
56.	34527	34811	285	+	50	Restriction endonucleases	VRR_NUC ; VRR-NUC domain	94	Salmonella phage BPS11Q3	97.85%	YP_009322848.1	10.9	0	9.96
57.	34843	35034	192	+	48	Hypothetical protein		63	Salmonella phage SETP7	100%	YP_008767205.1	7.0	0	9.73
58.	35031	35528	498	+	48	Hypothetical protein	HNH_3 ; HNH endonuclease	165	Salmonella phage vB_StyS-sam	96.97%	BBO65957.1	18.8	0	10.31
59.	35525	37990	2466	+	51	Putative helicase	Hom_end_hint ; Hom_end-associated Hint	821	Salmonella phage vB_SenS_PVP-SE2	97.56%	AST15461.1	92.5	0	8.80
60.	37987	38208	222	+	45	Hypothetical protein	DUF4140 ; N-terminal domain of unknown function (DUF4140)	73	Salmonella phage vB_SenS-Ent2	100%	YP_009009957.1	8.7	0	4.56
61.	38205	38297	93	+	43	Hypothetical protein	DUF6479 ; Family of unknown function (DUF6479)	30	Salmonella phage SG2	96.67%	ASZ76550.1	3.1	1	9.27
62.	40381	38327	2055	-	50	Tailspike protein	PhageP22-tail ; Salmonella phage P22 tail-spike	684	Salmonella phage wast	98.93%	QIN99606.1	72.9	0	4.93
63.	42361	40394	1968	-	49	Tail fiber protein	Phage-tail_3 ; Putative phage tail protein	655	Salmonella phage vB_SenS_PVP-SE2	99.54%	AST15460.1	72.4	0	5.21

Table S4: SGPC Promoter sequences and position on genome

Sr.no	Strand	Positions	Promoter Sequence	Type	Scores
1.	+	(11341..11366)	TTGACATTATATAGCCTCCGTATAAA	host	0.983
2.	+	(13994..14023)	TTTACATTGGCAATGAATAATTGGAATAAT	host	0.937
3.	+	(14907..14929)	AAATCAGCTTCACTAAAGACCAA	phage	0.804
4.	+	(16416..16445)	TAAAATATTTGTTGCTTAACCAAATATAAT	host	0.830
5.	+	(16427..16458)	TTGCTTAACCAAATATAATAGGGTACTATTAT	host	0.828
6.	-	(21440..21471)	TTGACTATGTGACAGATGGTAAAGTGTATGAT	host	0.925
7.	+	(21925..21932)	ACTAAATA	host	0.921
8.	-	(21932..21957)	TTGACTAGTAACATTAACCCTATTAT	host	0.989
9.	-	(22052..22077)	TTGACACATAGGAGTATTCCTAAGAT	host	0.977
10.	-	(25353..25381)	TAAAATGCACTTGTGTAACCCAATATAAT	host	0.914
11.	-	(25844..25872)	TTGACATTTTCATTTTTTTCGTGTAAGTT	host	0.836
12.	+	(25888..25916)	TTGATTTCCCATTATGCCGTGTGTATACT	host	0.922
13.	+	(26188..26219)	TTGACTTGCGCCGGACTTATTTTCAGTACTAT	host	0.906
14.	-	(26385..26411)	TTGACATGTGCTTTTCCTTTGTAGTGT	host	0.829
15.	+	(28901..28908)	CATAAATA	host	0.804
16.	+	(30964..30989)	TTGACAAGTTAATAGTACTCTATTAT	host	0.997
17.	+	(34780..34805)	TTGATAAATTAATAGAACACTATTAT	host	0.984
18.	-	(34805..34812)	ACTAAATA	host	0.961
19.	+	(35794..35822)	GTATACAGTAAACGAAAGCCATATATTAT	host	0.853
20.	-	(37962..37997)	TCATTATTCATCTCTTACCTCGTCTCTTCATATAAT	host	0.802
21.	+	(38264..38295)	TTTACTTACCGCTGCTGTGGTTCTGTTATCAT	host	0.826
22.	-	(38892..38919)	TTGCCCACGAAACAGTATTCACTAATAT	host	0.818

Table S5: Total Rho-independent terminators In SGPC predicted by Arnold

5' end position	Predicting program	Strand	Sequence	Free energy of stem loop region (Kcal/mol)
212	Rnamotif	+	GATGTCGTAACTGCGTCCGTACGGGCGCGccTTGTTGTTACGG	10.60
1045	Rnamotif	+	CCGGTTTCTGCGGTGAATACGCCTTTTTCTTGCGT	4.10
3086	Rnamotif	+	CGGTGTCACGGGCGGCATTATATGCCGCTATCTGTTCCTT	10.80
4099	Rnamotif	+	AATTTTGACAAGCCGTCGCCAGCGGCTTTTGACGTCGC	7.10
4107	Both	+	CAAGCCGTCGCCAGCGGCTTTTGACGTCGCTGagTTAGTTTTATCC	10.90
4475	Both	-	AAAACTGCTAAGGCGGAAGACAAGCATCCGCCTTTTATTTTCCA	9.70
4498	Rnamotif	+	GTCTTCCGCCTTAGCAGTTTTAGTGGCTGCTATTTTCCCCATCG	9.50
4539	Rnamotif	+	TCGCGATAAGGGCCGCCTCATGCCCGGCGGCTATGTTGTCGAA	12.70
4552	Rnamotif	+	CGCCTCATGCCCGGCGGCTATGTTGTCGaaTTTTTGTTTAGC	6.90
5091	Rnamotif	+	AGTTGCGTCAGGCGGGAACCACCCGCTTTTCCGGTGCG	8.80
6982	Rnamotif	-	CCTAAGAAAAAGGCCCCTTTCGGGGCCTTAGTTTCATTC	13.40
6983	Both	+	AATGAAACTAAGGCCCCGAAAGGGGCCTTTTTCTTAGGC	14.30
8135	Rnamotif	-	ACCATATAAGGGCGCGATGACCGCGCagTTTTACGTTCTT	8.80
11361	Rnamotif	-	GGTATACTCTTGAGGGACTTCGGTCCCTCTTTTCATTTATA	13.20
12431	Both	+	GATATCGCCAATAGTGGTAATTGCCATTATTTATTTCCTCA	6.00
14027	Both	-	AAGTAACGAAAGGCCCCTTCAAGGGGCCTTAATTTTGCCC	13.80
14028	Both	+	GGCAAAATTAAGGCCCCTTGAAGGGGCCTTTCGTTACTTA	13.80
16851	Both	-	GTAAACATAAAGGCCCCATAACGGGGCCcTTATGTCTCGTT	13.40
16853	Both	+	CGAGACATAAGGGCCCCGTTATGGGGCCTTTATGTTTACT	13.10
17617	Rnamotif	-	GGGAATCATTACGGGGGGTAATCCCGTTTACTTTTATC	6.90
20227	Both	-	CCTACCACCAAGCCCGCTTCTGTGGGCTTTTCTGTACAC	11.40
21974	Both	-	TAGCTCCTTTATCCCGGTAACTGACCCTACCGGGATTTTTTTTATCTG	13.30
23116	Both	-	AAATGTTAATCTCCGAAGGGTAAACCAACTTTCGGAgaTTTTTTATTGTG	10.70
23117	Both	+	ACAATAAAAAATCTCCGAAAGTTGGTTTACCCTTCGGAGATTAACATTTATGC	9.70
24275	Rnamotif	+	CCCTTTCGGCAGCGGGCCGCCGCgTTTTTCGATGCG	7.10
25571	Both	-	TAACGATTAGTGGGCAGGTTTTAGACCTGCCCTTTAGTTACCAG	15.40
26343	Both	-	TAGTGTTTAAGGCCCTGACGTTCGCGCGTTGGGGCTTTTCTTTTATTC	12.30
28587	Both	-	TTGGGGATACAGGGGCCTGATGGCCCCTTTCTTTTTACT	14.10
31683	Rnamotif	+	AATTTAACTAAGGCCCTTCATTGGGCCTTTTTACTAAGG	11.00
35199	Rnamotif	-	CGTAGACGAAAGCCCATGCCAGATGGTGGGCTTTTATTCTTAC	10.10
35299	Rnamotif	-	CTGAATCTGCTGGCTCCTTGTTGCGGGCCTTATATTATTTA	7.10
35936	Both	+	TATCTTAAGGGTTGGCGGGTACCTGTCGATTTTCCAAGGAA	6.20
38291	Both	-	TAAATAGTAAAGGCCCCGTAAGGGGCCTTAATTTATGAT	14.30
38292	Both	+	TCATAAATTAAGGCCCCTTACGGGGCCTTTACTATTTAT	13.40
39521	Rnamotif	+	CCGAATTTCCAGGGTAGAAGATATGCTTTGCCCTTTAGCTTCCGG	8.00
40164	Rnamotif	-	CAGTCTTACGTGATGCTGGTTTCTCCCCGGCATCTTTTAACTTTAC	12.00

Protein Superfamily	Index in Query Genome	Sequence Header
MCP	44	ORF 20
Portal	37	ORF27
TermL	36	ORF 28
MTP	53	ORF11
Sheath	NOT FOUND	
Ad1	48	ORF 16
Hc1	50	ORF 14
Ne1	51	ORF 13
Tc1	52	ORF P12

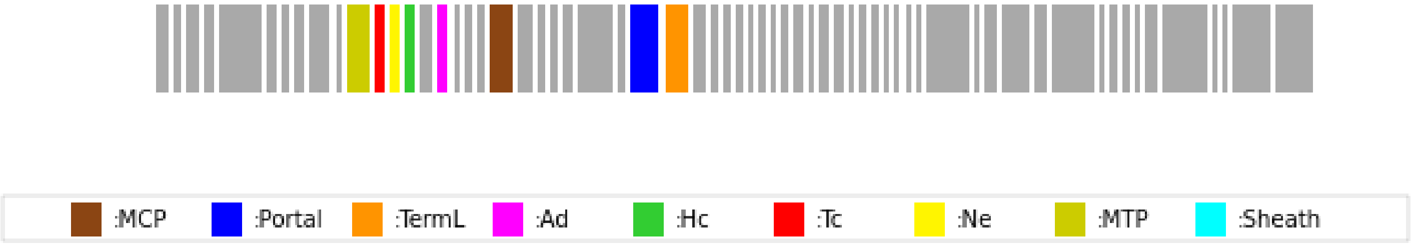

Figure S2: Neck Module and part of the Head and Tail Proteins arrangement suggesting SGPC belongs to Siphoviridae of Type1 (Cluster 5) , here MCP shows major capsid protein, Portal proteins, TermL refers to Terminal proteins, Ad for adapters, Hc for head closure, Tc for Tail completion ,Ne for neck protein and MTP refers to major tail protein
